# DGC/Zeta as A New Strategy to Improve Clinical Outcome in Male Factor Infertility Patients following Intracytoplasmic Sperm Injection: A Randomized, Single-Blind, Clinical Trial 

**DOI:** 10.22074/cellj.2020.6525

**Published:** 2019-09-08

**Authors:** Nazanin Karimi, Homa Mohseni Kouchesfahani, Mohammad Hossein Nasr-Esfahani, Marziyeh Tavalaee, Abdolhossein Shahverdi, Hamid Choobineh

**Affiliations:** 1 Department of Animal Biology, Faculty of Biological Sciences, Kharazmi University, Tehran, Iran; 2 Department of Reproductive Biotechnology, Reproductive Biomedicine Research Center, Royan Institute for Biotechnology, ACECR, Isfahan, Iran; 3 Reproductive Epidemiology Research Center, Royan Institute for Reproductive Biomedicine, ACECR, Tehran, Iran; 4 Department of Embryology, Reproductive Biomedicine Research Center, Royan Institute for Reproductive Biomedicine, ACECR, Tehran, Iran; 5 School of Allied Medical Science, Tehran University of Medical Sciences, Tehran, Iran

**Keywords:** DGC/Zeta, Embryo Quality, Fertilization, Pregnancy

## Abstract

**Objective:**

The aim of this blind randomised clinical trial study was to assess the clinical efficiency of combined density gradient centrifugation/Zeta (DGC/Zeta) sperm selection procedure compared to conventional DGC in infertile men candidates for intracytoplasmic sperm injection (ICSI). The literature shows that DGC/Zeta is more effective compared to DGC alone in selection of sperms with normal chromatin and improves the clinical outcome of the ICSI procedure. Therefore, this study re-evaluates the efficiency of DGC/Zeta in improving the clinical outcomes of ICSI in an independent clinical setting.

**Materials and Methods:**

In this randomized, single-blind, clinical trial, a total of 240 couples with male factor infertility and at least one abnormal sperm parameter were informed regarding the study and 220 participated. Based on inclusion and exclusion criteria, 103 and 102 couples were randomly allocated into the DGC/Zeta and DGC groups, respectively. ICSI outcomes were followed and compared between the two groups.

**Results:**

Although there was no significant difference in fertilization rate (P=0.67) between the DGC/Zeta and DGC groups, mean percentage of good embryo quality (P=0.04), good blastocysts quality (P=0.049), expanded blastocysts (P=0.007), chemical pregnancies (P=0.005) and clinical pregnancies (P=0.007) were significantly higher in the DGC/ Zeta group compared to DGC. In addition, implantation rate was insignificantly higher in DGC/Zeta compared to DGC (P=0.17).

**Conclusion:**

This is the second independent study showing combined DGC/Zeta procedure improves ICSI outcomes, especially the pregnancy rate, compared to the classical DGC procedure and this is likely related to the improved quality of sperm selected by the DGC/Zeta procedure (Registration number: IRCT20180628040270N1).

## Introduction

Preparation of a sperm population with high chromatin normality is a basic parameter which is strived for in intracytoplasmic sperm injection (ICSI) procedures. Today, selection of sperm for ICSI is based on sperm morphology and viability (1). The percentage of sperm with strict normal morphology is believed to be correlated with percentage of sperm with normal DNA content, however, this concept has been highly debated (2, 3). There are reports that the percentage of sperm with fragmented DNA but normal morphology increases in subfertile/infertile individuals compared to fertile men (4, 5). In this regrade, previous studies have showed that the percentage of DNA fragmentation in sperm from neat semen of infertile men with normal and abnormal semen parameters are around 30 and, 20-40%, respectively. Sperm with fragmented DNA may fertilize an oocyte, but it has a reduce chance of pre- or post-implantation development (6).

A recent meta-analysis has suggested that assessment of sperm DNA fragmentation is beneficial in predicting male fertility (7). A plethora of studies have also concluded that routine sperm preparation procedures such as swim up, and density gradient centrifugation (DGC) alongside novel sperm preparation procedures based on sperm molecular and cellular characteristics can separate a higher percentage of normal sperm with intact DNA compared to routine sperm preparation procedures, especially in infertile couples with severe male factor infertility (1, 8, 9).

Sperm preparation based on surface electrical charge or Zeta potential has been introduced as one of the novel sperm preparation procedures. This procedure, was initially introduced by Chan et al. (10) who showed Zeta potential is an effective and feasible procedure for selecting of sperm with intact DNA structure. Sperm with a high level of surface negative electrical charge are more mature and several studies have shown that the percentage of sperm with fragmented DNA were significantly lower in sperm selected in this way. Subsequent studies verified that sperm selected based on Zeta potential have a higher chance of having normal intact chromatin (11, 12). Later, this procedure was compared to another novel sperm preparation method based on hyaluronic acid binding and the results showed that although both novel sperm preparation procedures can improve the percentage of sperm with DNAfragmentation, the Zeta method was more efficient (13). In addition, it was shown that combined DGC/Zeta procedures boost the quality of the sperm selected for ICSI and lead to higher clinical pregnancy rates per embryo transfer cycle (14, 15). Considering the need for further clinical studies to evaluate the impact of sperm quality on assisted reproductive technology (ART) outcomes, wider multi-center randomized studies are required to verify the beneficial effects of DGC/Zeta sperm selection. Therefore, the aim of this blind randomised clinical trial was to evaluate the effectiveness of the DGC/ Zeta procedure in improving clinical outcomes in infertile men in an independent center. 

## Materials and Methods

### Patients

All procedures performed involving human participants were in accordance with the ethical standards of the institutional research committee and with the 1975 Helsinki declaration and its later amendments or comparable standards. We performed a randomized, single-blind, clinical trial study from April 2015 till August 2017 at Rouyesh Infertility and Fertility Center belonging to the Red Crescent (IRCT20180628040270N1), after approval by the Ethical Committee of Tehran Royan Institute [Code No: IR.ACECR.Royan.REC.1396.253]. 

All participants received a complete explanation of the trial prior to the start of the study. They were especially ensured that their semen samples would not be exposed to chemical agents. Following voluntary completion of the questionnaire, the couples signed informed consent forms. Eligible individuals were assured of confidentiality and anonymity, and that their decision to participate in, or withdraw from the study would not impact their current or future relation with the clinic or their future treatment. In this parallel blind randomized clinical trial, 205 candidate couples for ICSI cycles were randomly assigned to the DGC or DGC/Zeta groups based on a computer generated random table. 

#### Inclusion criteria

Females were between 20 and 40 years old, with no report of endometriosis or polycystic ovaries. Presence of at least 2 to 3 follicles more than18 mm in diameters with suitable endometrium for embryo transfer in their last ultrasound before administration of human chorionic gonadotropin (hCG). Only couples with male factor infertility and at least one abnormal sperm parameter (sperm motility, concentration and morphology) below world health organization (WHO, 2010) criteria were included in this study (16). 

#### Exclusion criteria

Couples whose rate of oocytes with abnormal features (without polar body, germinal vesicle, granularity, refractile bodies, fragmented, or degenerated polar bodies) exceeded 10%, were excluded from the study. Couples that did not meet the above-mentioned ultrasound criteria were also exclude from the study. As were infertile men with varicocele. 

#### Sperm preparation using density gradient centrifugation 

A two-layer density gradient system (40 and 80%) was prepared using PureSperm (Nidacon, Göteborg, Sweden). Following semen liquefaction, each semen sample was placed on the gradients and centrifuged at 300 g for 20 minutes. Then, the pellet was collected and re-suspend into 5mlof sperm processing medium supplemented with 10% human serum albumin (HSA, Octalbin, Switzerland). The sperm suspension was then centrifuged at 300 g for 7 minutes. For insemination; the resultant pellet was diluted into 0.3 mL of sperm processing medium containing10% HAS albumin (14). 

#### Density gradient centrifugation/Zeta procedure 

For DGC/Zeta procedure, sperm pellets were washed with sperm processing media without HSA, and subsequently diluted in 4 ml sperm processing media without HSA, immediately after DGC. Subsequently, the tube was exposed to a positive charge using a rubber latex tube (14). The tube was then removed from the latex tube and held by the cap for one minute to provide the time needed for the sperm with adequate negative charge to attach to the charged tube. Then the sperm processing media containing unattached sperm was withdrawn from the tube and the tube was washed with sperm processing medium containing HSA in order to detach the attached sperm. Ultimately, the content of each tube was centrifuged and the pellet was diluted in sperm processing media with HSA and used for ICSI. To reduce inter-sample variation, a single trained individual carried out all the procedures for sperm processing. The embryologist who inseminated the sperm was blind to the allocation of the sperm to the two groups; DGC and DGC/Zeta. 

#### Intracytoplasmic sperm injection procedure

Stimulation and ovum pick up procedures were performed base on a single standard protocol for all cases (17). Around 16-18 hours post-ICSI, the presence of male and female pronuclei was considered as a sign of successful fertilization and the rate of fertilization was calculated as the percentage of injected oocytes that became fertilized. An embryo was selected and considered to be a top quality embryo if there were six to eight blastomeres on day 3 with fragmentation less than 25% and the absence of multi nucleated blastomeres at any stage of early development. The percentage of top quality embryos was defined as the number of top embryos obtained from the total number of cleaved embryos. Procedures of embryo transfer were similar in both groups and were carried out by an embryologist who was not aware of the design of the clinical trial. The embryologist was asked to select the best embryos for transfer and a minimum of one and a maximum of three embryos were transferred. Based on the internal policy of the center, individuals under the age of 35 can receive a maximum of 2 embryos whileindividualsover35 were allowed to request for the transfer of a maximum of 3 embryos if two top quality embryos are not available. Blastocyst quality was assessed on day 5 (18). All embryos were transferred fresh. Chemical and clinical pregnancy were defined as ß-hCG levels higher than 10 IU and the presence of a gestational sac, 5 weeks after embryo transfer, respectively. 

#### Statistical analysis

SPSS for Windows Version 18 (SPSS Inc., Chicago, IL, USA) was used for all statistical analyses. Data are presented as means ± SEM for continuous variables. Independent-samples t test was used for comparisons of couples’ age and sperm parameters between DGC/ Zeta and DGC groups (Table 1). Independent student's t test and Chi-square carried out for statically analyzing was used for comparisons of fertilization, good-quality embryo, pregnancy, implantation, and miscarriage (Table 2). The value of P<0.05 was considered statistically significant. This clinical trial study was a continuation of the Nasr-Esfahani group study (15). To detect the effect of DGC/Zeta on clinical outcome in male factor infertility patients following ICSI which is in agreement with the study of Nasr Esfahani et al. (15) with a power of 80%, a sample size of 103 patients per group was necessary, given an anticipated dropout rate of 10%. 

**Table 1:** Comparison of couples age, and sperm parameters between density gradient centrifugation (DGC)/Zeta and DGC groups


Parameters	DGC/Zeta	DGC	P value

Male age (Y)	36.40 ± 3.28	35.87 ± 2.35	0.25^NS^
Female age (Y)	31.25 ± 0.44	32.04 ± 0.58	0.23^NS^
Sperm concentration (10^6^/ml)	34.44 ± 3.41	35.22 ± 3.20	0.38^NS^
Total sperm motility (%)	37.74 ± 1.41	38.21 ±1.37	0.90^NS^
Progressive motility (%)	14.83 ± 1.61	15.01 ± 1.48	0.54^NS^
Sperm normal morphology (%)	3.20 ± 0.41	4.04 ± 0.65	0.11^NS^


Data are presented as mean ± SEM and analyzed by independent-samples t test. Asterisk indicate significant difference; *; P<0/05, and NS; Non significant.

**Table 2:** Comparison of clinical outcomes between density gradient centrifugation (DGC)/Zeta and DGC groups


Parameters	DGC/Zeta n=103	DGC n=102	P value

Number of oocyte retrievals	6.54 ± 0.35	7.12 ± 0.30	0.15^NS^
Fertilization rate (%)	64.75 ± 1.67	58.88 ± 1.83	0.67^NS^
Number of embryos transferred	2.40 ± 0.11	2.28 ± 0.10	0.80^NS^
Good quality of embryo at day 3 (%)	41.89 ± 2.01	41.89 ± 2.01	0.04*
Blastocyst formation rate on day 5 (%)	41.5 ± 1.53	37.84 ± 1.71	0.51^NS^
Good quality blastocyst (%)	33.69 ± 1.22	23.86 ± 1.51	0.049**
Expand blastocyst (%)	48.2 ± 2.11	39.24 ± 2.75	0.007**
Hatching blastocyst (%)	1.2 ± 0.82	0.4 ± 0.68	0.005**
Clinical pregnancy rate (%)	36/103 (35%)	21/102 (20.68%)	0.007**
Implantation rate (%)	21/103 (20.48%)	12/102 (11.42%)	0.17^NS^
Miscarriages rate (%)	5/56 (8.92%)	6/31 (19.35%)	0.04*
Chemical pregnancy (%)	44/103 (42.71%)	22/102 (21.56%)	0.005**


Data are presented as mean ± SEM and analyzed by Independent studentʼs t test and Chi-square. Asterisks indicate significant difference; **; P<0/01 , *; P<0/05, and NS; Non significant.

### Results

In this randomized clinical trial, a total of 240 infertile couples were recruited and 220 couples agreed to partake in this study. These couples were randomly divided into two groups based on the randomization table generated by a computer into the DGC/Zeta or DGC groups. Of the 220 infertile couples, 7 couples were excluded from the study based on exclusion criteria (3 and 4 couples in the DGC/ Zeta and DGC groups, respectively). Of the 213 remaining couples 8 couples decided to leave the study for personal reasons. Finally, of the 205 remaining couples 103 and 102 belonged to DGC/Zeta and DGC groups, respectively. Baseline characteristics of the DGC/Zeta and DGC groups including male age (P=0.25), female age (P=0.23) semen parameters including [sperm concentration (P=0.38), total motility (P=0.9) and normal morphology (P=0.11)] were analyzed and compared between the two groups. These were found to be similar in both groups and no significant differences were observed (Table 1). 

#### The clinical outcomes 

The clinical outcomes of a total 205 cycles in two groups were evaluated and compared (Table 2). The mean number of retrieved oocytes and transferred embryos in the two groups were similar without any significant differences. An obvious drift towards a superior fertilization rate was seen in the DGC/Zeta procedure compared to DGC alone (64.75 ± 1.67 vs. 58.88 ± 1.83, P=0.67). Although, there was no statistically significant difference in the mean number of embryos transferred between the two groups (P=0.8), but mean percentage of good embryo quality at day 3 (41.89 ± 2.01 vs. 30.64 ± 3.51, P=0.04), good quality of blastocysts (33.69 ± 1.22 vs. 23.86 ± 1.51, P=0.049), expanded blastocysts (48.2 ± 2.11 vs. 39.24 ± 2.75, P=0.007), and mean of hatching blastocysts (1.2 ± 0.82 vs. 0.4 ± 0.68, P=0.005) were significantly higher in the DGC/Zeta group compared to the DGC group. The percentage of chemical (P=0.005) and clinical (P=0.007) pregnancy rates in the DGC/Zeta group were 42.47 and 35.03, respectively. However these rates were 21.10% and 20.43% respectively in the DGC group. The mean percentage of implantation rate were insignificantly (P=0.17) higher in the DGC/Zeta group (20.80) compared to the DGC group (11.96). While the mean percentage of missed spontaneous abortion/missed miscarriages were significantly (P=0.04) lower in the DGC/Zeta group (8.9) compared to the DGC group (19.01). 

### Discussion

Numerous methods have been developed to eliminate morphologically normal sperm with damaged DNA from being inseminated during the ICSI procedure, which is a shortcoming of conventional ICSI procedures. Each of these approaches has advantages and disadvantage which have been covered by extensive reviews in this filed (1, 8, 9). The outer surface of the sperm plasma membrane is rich in sialic acids. These sialic acids are responsible for the membrane’s negative electrical charge of around -16 to -20 mV called the "Zeta potential" or electrokinetic potential (19, 14). Sperm selection based on Zeta potential is a new strategy in order to acquire functional sperm in a manner that optimizes sperm recovery rates specially of sperm with normal DNA integrity to improve ICSI outcomes (20, 21). 

Despite clear evidence showing benefits of selecting sperm based on Zeta potential, low enthusiasm for the implementation of this technique is due to the limited number of clinical trials. Ainsworth et al. (22-24) and Fleming et al. (25) introduced a device based on Zeta potential or electrophoresis and their preliminary studies showed that ICSI outcomes can be improved by this approach. In this regard, the first pregnancy reported using the electrophoresis method in infertile couples with previous repeated failed fertilization, and high sperm DNA fragmentation (22). In addition, Fleming et al. (25) compared clinical outcomes of ICSI or IVF between the electrophoretic method and the DGC procedure, and concluded that the mean fertilization rate, and embryo quality were similar between the two groups. Then, they reported that the electrophoretic method can be harmful for sperm motility. Unlike the electrophoretic method, the Zeta method is simple, low cost, fast, and no chemicals are used during the preparation of the sperm. In this regard, the Nasr-Esfahani et al. (15), by inducing positive charge on the surface of a tube showed that ICSI outcome, especially clinical pregnancy rate can be improved by this technique compared to DGC alone. Based on their experience, we also decided to assess the efficiency of this technique in a sister clinic in different location independently of this group but through their collaboration and with the transfer of experience. Thus, following randomization, a total of 103 and 102 infertile couples were allocated to the DGC/Zeta and DGC groups, respectively and the clinical outcomes were evaluated. In this study, higher rates of good embryo quality, blastocysts, expanded blastocysts, hatching blastocysts, chemical and clinical pregnancy were seen in the DGC/Zeta group. In addition, the results of this study also revealed that the selection of sperm through the DGC/Zeta procedure did not significantly affect the fertilization rate but significantly improves embryo quality. This is consistent with the results of previous studies suggesting that sperm DNA damage does not necessarily preclude sperm from participating in the process of fertilization but can significantly affect the embryo quality especially during maternal- embryonic genomic transition (26). These results are in agreement with a previous randomized trial study by Nasr Esfahani et al. (15) and represented an improvement in clinical outcomes after injection of sperm with DGC/ Zeta processing. Major causes of significant difference in clinical consequences between the two procedures, (DGC/ Zeta vs. DGC alone), may be due to the ability of DGC/ Zeta for selecting mature sperm with lower DNA damage compared to DGC alone. Indeed, a high rate of sperm DNA damage has been associated with reduced clinical outcomes following assisted reproduction, increased time to conception and high rate of abortion (11, 26-29).

## Conclusion

The results of this blind clinical trial along with other reports in the literature reveal that selection of sperm based on Zeta is able to recover a population of mature sperm with intact DNA and eliminate sperm with a high degree of DNA fragmentation. Therefore, the improved efficacy should be particularly valuable in ART. Accordingly, we recommend specialists working in field of assisted reproduction to evaluate the capacity of Zeta procedure especially for couples with previous implantation failures. However, use of a device for the selection of sperm based on Zeta potential may even further improve the efficiency of clinical outcomes post ICSI and reduce variations between different studies.
